# First Detection and Delimiting Survey of the Emerald Ash Borer (*Agrilus planipennis* Fairmaire, 1888 (Coleoptera: Buprestidae)) in Slovakia

**DOI:** 10.3390/insects17101019

**Published:** 2026-10-02

**Authors:** Andrej Gubka, Marcel Dubec, Christo Nikolov, Kristína Darnadyová, Ivana Kurhajcová, Maroš Lučaj, Juraj Galko, Andrej Kunca, Michal Lalík, Jozef Vakula, Slavomír Rell, Valéria Longauerová, Roman Leontovyč, Milan Zúbrik

**Affiliations:** 1 National Forest Centre, T. G. Masaryka 2175/22, 960 01 Zvolen, Slovakia; 2 Laboratory of Quarantine Diagnostics, Central Control and Testing Institute in Agriculture, Letná 3, 041 39 Košice, Slovakia; 3 Central Control and Testing Institute in Agriculture, Department of Plant Protection, Matúškova 21, 833 16 Bratislava, Slovakia

**Keywords:** *Agrilus planipennis*, emerald ash borer, first record, Slovakia, invasive alien species, quarantine pest, *Fraxinus*, delimiting survey

## Abstract

The emerald ash borer, *Agrilus planipennis* Fairmaire, 1888 (Coleoptera: Buprestidae), is an invasive pest in Europe and North America, responsible for extensive mortality of ash (*Fraxinus* spp.). This study reports its first confirmed occurrence in Slovakia and summarises the subsequent delimiting survey. On 25 June 2026, specimens morphologically resembling *A. planipennis* were collected from a monitoring trap in a park in Streda nad Bodrogom, south-eastern Slovakia. Eighteen adults were identified as *A. planipennis* by the national quarantine diagnostics laboratory, with confirmation by the European Union Reference Laboratory in France. Initial field inspections revealed characteristic galleries and D-shaped exit holes on ash trees at the detection site, in nearby forests, and along roads within approximately 7 km. By 31 July 2026, 2250 survey points had been recorded, of which 149 showed evidence of *A. planipennis*, including adults, larvae, galleries, or exit holes. Old galleries together with living larvae indicate that the pest had probably been established in Slovakia for several years before detection in 2026. Symptom assessment was complicated by widespread ash dieback caused by *Hymenoscyphus* fraxineus (T. Kowalski) Baral, Queloz & Hosoya, 2014.

## Simple Summary

Emerald ash borer is an invasive beetle that attacks ash trees and is a regulated quarantine pest in the European Union. This study documents its first confirmed occurrence in Slovakia and the survey undertaken to determine how widely it had spread. Adults were first found in a monitoring trap in Streda nad Bodrogom in south-eastern Slovakia on 25 June 2026. Laboratory identification was performed by the Laboratory of Quarantine Diagnostics in Košice, Slovakia and confirmed by the European Union Reference Laboratory in France. Follow-up inspections found characteristic damage on ash trees near the detection site and at more distant locations up to 50 km away. By 31 July 2026, 2250 survey points had been recorded, with evidence of emerald ash borer at 149 points. Old galleries together with living larvae of this pest suggest that the beetle had been present in Slovakia for several years before it was detected in 2026. Survey work was complicated by widespread ash dieback and by damage caused by other insects that can resemble emerald ash borer symptoms. Continued surveillance is needed to define its distribution and detect isolated infestations.

## 1. Introduction

The emerald ash borer (EAB), *Agrilus planipennis* Fairmaire, 1888 (Coleoptera: Buprestidae), is native to East Asia, where it primarily attacks indigenous ash species adapted to its presence [1,2]. In Europe, its development has been confirmed on European ash (*Fraxinus excelsior* L., 1753), narrow-leaved ash (*F. angustifolia* Vahl, 1804), manna ash (*F. ornus* L., 1753), and green ash (*F. pennsylvanica* Marsh., 1785), the latter being among the most susceptible hosts [3,4]. Outside the genus *Fraxinus*, development of this species has also been recorded on *Chionanthus virginicus* L., 1753 (Oleaceae) [5].

The species usually has one generation per year, but development may take two years in cooler conditions or in vigorous trees [6]. Adults emerge in May or June and may remain active until September. Females lay 40–90 eggs in bark crevices. Larvae bore into the phloem and cambial zone, where they form serpentine galleries. Adults leave the tree through characteristic D-shaped exit holes approximately 3–4 mm wide [2,7]. Early infestation is difficult to detect because the larvae are concealed and the crown may remain free of conspicuous symptoms [8,9]. The beetle spreads locally by flight, whereas distant outbreaks arise primarily through the transport of infested firewood, roundwood, and wood packaging material [1,7]. Owing to its high invasive potential, EAB is regulated in the EU as a quarantine pest and has also been listed as a priority pest since 2019 [9,10,11]. In Europe, the species was first identified in 2007 from specimens collected in Moscow between 2003 and 2006; untreated wood from Asia was considered the likely source [1,12,13]. It subsequently spread through European Russia, where it had been recorded in 18 administrative regions by 2022 [1,14]. It was recorded in Ukraine in 2019 and in Belarus in June 2025 [15,16]. The first confirmed record of EAB in EU territory was made in June 2026, when an adult was trapped in the Hungarian village of Beregsurány near the Ukrainian border [17]. This study reports the first confirmed occurrence of *A. planipennis* in Slovakia and describes the subsequent delimiting survey.

The Central Control and Testing Institute in Agriculture (CCTIA) is the main authority responsible for phytosanitary controls, monitoring, and measures against quarantine pests in Slovakia. Its activities include the inspection of plants, plant products, and other regulated articles during cultivation, trade, and import from non-EU countries. Since 2015, CCTIA has cooperated with the Forest Protection Service of the National Forest Centre (NFC) in monitoring quarantine organisms, particularly in forest stands, and in educational and outreach activities. Professionals and the general public were informed about *A. planipennis* through conferences, training courses, articles, and information materials. The species was also included in the online atlases www.forestpests.eu (accessed on 29 September 2026) [18] and www.skodcoviadrevin.sk (accessed on 29 September 2026) [19], which provide photographic documentation, descriptions, and the option to submit photographs for identification. These platforms also supported public participation in searching for the species through citizen science. Slovak experts have also participated in EFSA and EPPO working groups, contributing to national preparedness for the possible occurrence of EAB.

In 2025, a revised national contingency plan for *A. planipennis* was approved [20]. A simulation exercise was subsequently held in the High Tatras to test procedures for responding to its detection.

## 2. Materials and Methods

### 2.1. Pheromone Trap Monitoring

Pheromone traps were installed annually from 2023 at selected locations in Slovakia, with approximately 11 traps deployed per year. Green multiple-funnel traps (Synergy Semiochemicals Corp., Delta, BC, Canada), baited with ECONEX AGRILUS SPP 60 DAYS pheromone diffusers (Sanidad Agrícola ECONEX, S.L., Siscar-Santomera, Murcia, Spain), were used. The traps were checked at two-week intervals, and the catches were sent for diagnosis to the Central Control and Testing Institute in Agriculture, Laboratory of Quarantine Diagnostics, Košice, Slovakia.

### 2.2. Ground-Based Monitoring—Delimiting Survey

In July 2026, following the first record of *A. planipennis* we conducted survey using prepared maps showing municipal cadastral boundaries and forest stands containing *Fraxinus* species. Approximately 20 CCTIA plant health inspectors received field training provided by staff of the NFC Forest Protection Service. The training included demonstrations of characteristic EAB damage symptoms in nearby infested stands and practical identification of infested trees. Particular attention was given to distinguishing EAB signs from similar symptoms and other potential sources of misidentification. Inspectors were also trained in the use of the mobile survey application and in standardized recording of survey data. The combination of field training, standardized data recording, and photographic documentation of positive records was intended to improve the consistency of the survey data and reduce the risk of misidentification. Inspectors were divided into small teams of two or three, with each team assigned a municipality’s cadastral area to inspect each day. Visual inspections prioritised declining, dying, or recently dead ash trees, although asymptomatic ash trees were also examined. Attention focused mainly on trunks up to approximately 2–3 m above ground level, where inspectors searched for characteristic D-shaped exit holes approximately 3–4 mm in diameter. This trunk-focused approach was chosen to maximize the area covered within the available time and personnel, as systematic crown inspection would have been considerably more time-consuming and often required specialized access. Crown inspection was therefore not conducted routinely, although upper parts of selected trees were examined when feasible. Where suspicious holes were found, bark was removed to check for typical serpentine larval galleries in the phloem and cambium, or for larvae themselves. A single characteristic exit hole, gallery, or EAB specimen was considered sufficient evidence that a site was infested. The geographic coordinates and infestation status of each surveyed location were recorded in the mobile application (Agrilus-map) as either positive or negative, and positive records were supported by photographic documentation for verification. The mobile application (Agrilus-map) was developed by NFC specifically for the EAB survey to coordinate the delimiting survey and standardize field data collection. Collected larvae and adults were placed in plastic tubes and transported to the CCTIA Laboratory of Quarantine Diagnostics in Košice for laboratory examination. An example of the mobile application interface and recorded survey points is shown in Figure 1.

## 3. Results

On 25 June 2026, inspectors checking a trap installed by CCTIA in a park in Streda nad Bodrogom (48.369778° N, 21.752663° E), near the Hungarian border, found a sample containing suspected *A. planipennis* specimens. It was sent to the CCTIA Laboratory of Quarantine Diagnostics in Košice. On 3 July 2026, the laboratory confirmed 18 specimens of *A. planipennis*, representing the first record of the species in Slovakia. The sample was then sent to the European Union Reference Laboratory in France for confirmation, and the finding was confirmed on 20 July 2026. The pheromone trap at the detection site and the laboratory-confirmed adults are shown in Figure 2 and Figure 3, respectively.

On 7–8 July 2026, NFC and CCTIA conducted an initial survey to determine whether the finding was limited to the trap or whether trees were also infested. Exit holes and galleries were found on a tree in the park, in nearby forest stands, and on roadside ash trees approximately 7 km from the trapping site. These findings prompted several rounds of an extensive delimiting survey in the surrounding area.

From 14 to 17 July, during this survey, 573 sites containing individual ash trees or groups of ash trees were inspected in forests and urban places, producing 107 positive records.

A second survey round took place from 20 to 24 July, during which 1457 sites with individual ash trees or groups of ash trees were inspected. Only 26 sites were positive.

From 27 to 31 July, 220 sites were inspected, of which 16 were positive (Table 1). Exit holes, larvae, and both live and dead adult beetles were found. Some larvae were parasitised by representatives of the order Hymenoptera, like *Spathius* spp. The parasitoids were collected but have not yet been identified to species.

In parallel with the main survey, several additional sites were inspected, and some sites with inconclusive positive findings were revisited. The spatial distribution of positive and negative survey records is shown in Figure 4. Characteristic galleries and exit holes recorded during the survey are shown in Figure 5 and Figure 6. Examples of an older heavily infested tree and potentially confounding bark-beetle exit holes are shown in Figure 7 and Figure 8, respectively.

## 4. Discussion

Despite annual ground surveys of forest stands and pheromone-trap monitoring throughout Slovakia, *A. planipennis* was not detected before 2026. One possible reason is that early infestations may remain difficult to detect because EAB can initially colonise smaller branches in the crown [21]. Traps installed annually along the Ukrainian border yielded no captures until 2026. In 2025, a field survey was even conducted in one of the ash stands now known to be infested, yet no signs of infestation were detected. This negative result illustrates the difficulty of detecting low-density EAB populations. Siegert et al. [22] reported that the onset of ash mortality at newly infested sites may lag EAB establishment by several years, which can substantially delay visual detection. These findings also highlight the value of timely information on pest spread in neighbouring countries, which can help focus surveillance on areas of highest potential risk. Following trap detections in Hungary and Slovakia, an extensive delimiting survey was launched in south-eastern Slovakia and confirmed that EAB was established and reproducing in the area. Given the extent of infestation and the presence of older galleries together with living larvae, establishment is likely to have preceded the first confirmed record by several years.

Field identification was further complicated by other causes of damage and decline in ash. Many trees in the area were infested by *Hylesinus varius* (Fabricius, 1775) and *Hylesinus crenatus* (Fabricius 1787). Adults of these species commonly feed on ash trunks [23,24], producing round exit holes similar in size to those of *A. planipennis*. The delimiting survey was also complicated by the widespread decline in ash in Slovakia caused by *Hymenoscyphus fraxineus* [25]. Ash affected by this pathogen can display dieback symptoms similar to those associated with EAB infestation [2,26]. Many symptomatic trees inspected during the survey also showed signs consistent with ash dieback, and in several cases severely declining trees showed no direct evidence of EAB infestation. General crown decline was therefore of limited diagnostic value, reinforcing the need to rely on characteristic EAB signs such as D-shaped exit holes, galleries, larvae, or adults.

Following confirmation of EAB, an extensive delimiting survey identified a main area of infestation together with several spatially isolated positive records. By 31 July, the mobile application contained 2250 survey points, of which 149 were positive based on the presence of adults, larvae, galleries, or exit holes of *A. planipennis*. No evidence of EAB was recorded at the remaining 2101 points. The area with the highest concentration of positive records was roughly estimated, as of 31 July 2026, to cover more than 60,000 ha in the Zemplín Lowland of south-eastern Slovakia. Additional positive records occurred outside this area, including several approximately 10 km away and the most distant approximately 24 km from the main infested area. However, the isolated Slovak records should not yet be interpreted as established satellite populations. Rather, they indicate that delimiting surveys should extend beyond the apparent continuous infestation zone and include targeted surveillance for spatially isolated populations. Particular attention may be warranted at sites associated with movement or storage of ash material and along major transport routes, because human-assisted transport has been shown to contribute to the establishment of distant EAB infestations [1,7].

At present, the preparation and designation of an infested zone and a monitoring zones are designed in accordance with the guidelines set out in the contingency plan for the occurrence of *A. planipennis* in Slovakia [20]. This includes the designation of an infested zone extending at least 100 m around the recorded occurrence of *A. planipennis*, followed by the implementation of appropriate phytosanitary measures. A buffer zone extending 10 km around the infested zone is also to be designated, with intensive monitoring planned within this area. In addition, special phytosanitary requirements apply to specified host material originating within 100 km of the outer boundary of the infested zone.

## 5. Conclusions

Based on the presence of older galleries, living larvae, and the extent of infestation, EAB was likely established in Slovakia several years before its first detection in 2026. The delimiting survey revealed a main infested area in south-eastern Slovakia together with several spatially isolated positive records, indicating the need for continued surveillance beyond the apparent continuous infestation zone. Parasitoids, including *Spathius* spp., were also recorded, but their identity and potential role in EAB population dynamics require further investigation. The mobile application facilitated survey coordination and standardized data collection. Continued surveillance, particularly for isolated and low-density infestations, will be important for improving knowledge of the current distribution and further spread of EAB in Central Europe.

## Figures and Tables

**Figure 1:**
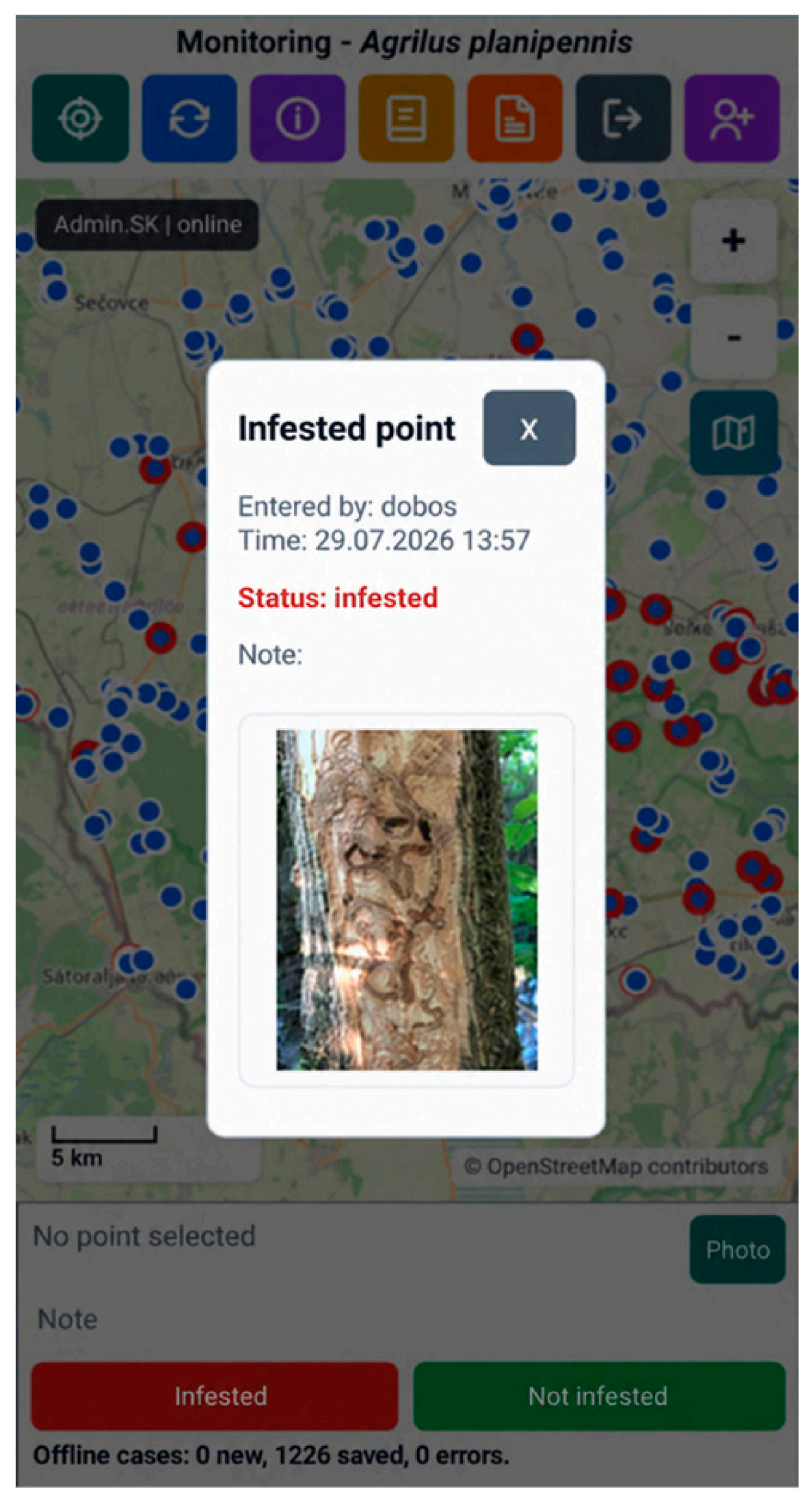
Screenshot of the mobile application used during the EAB delimiting survey. The map displays recorded survey points, with red and blue dots indicating positive and negative records, respectively. The detail view of a positive record includes the user who entered the record, the date and time of entry, infestation status, and an uploaded photograph documenting characteristic EAB galleries at the surveyed point. Red text indicates a positive (infested) status.

**Figure 2:**
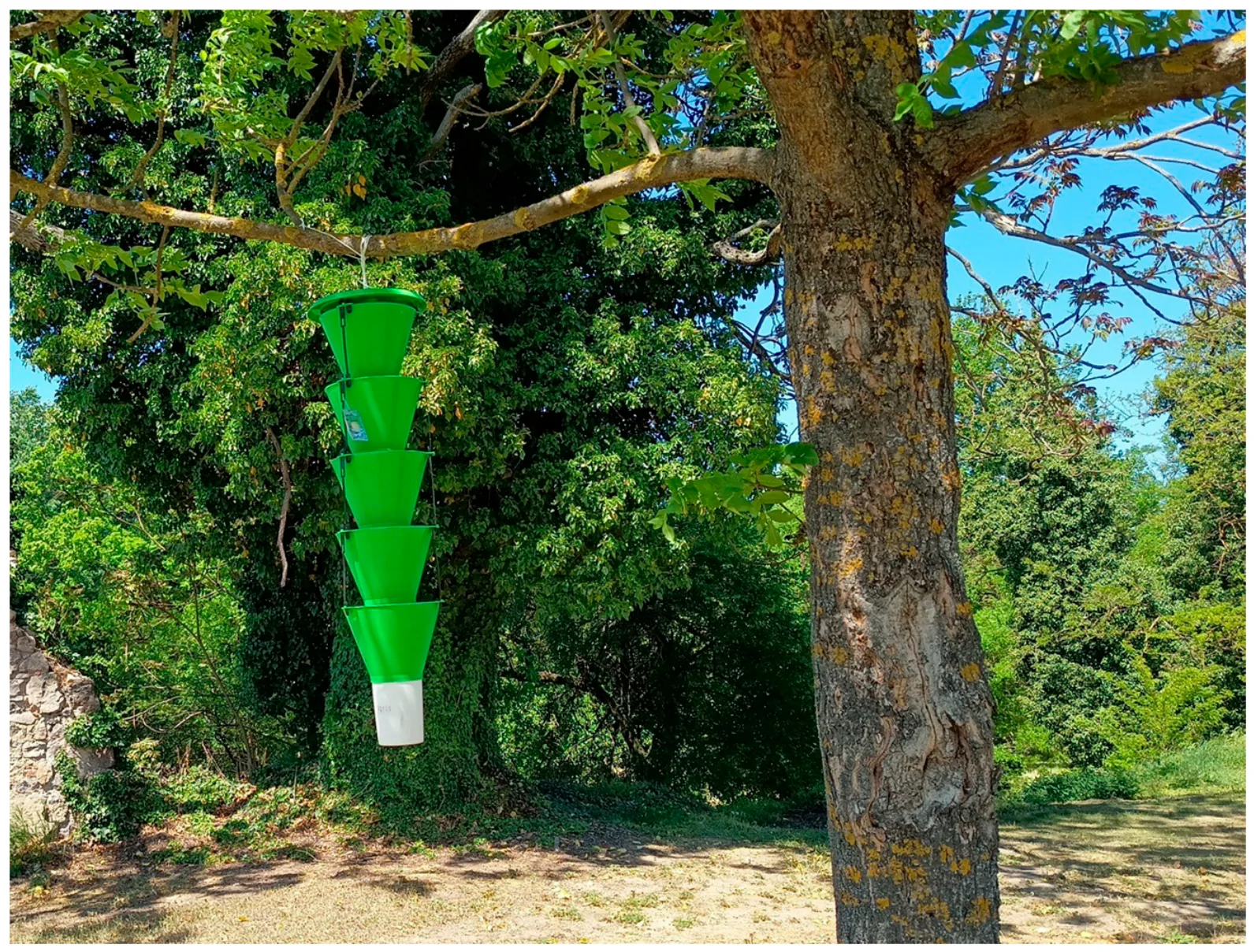
Pheromone trap installed in the park in Streda nad Bodrogom where EAB was first captured (photo: Martin Doboš).

**Figure 3:**
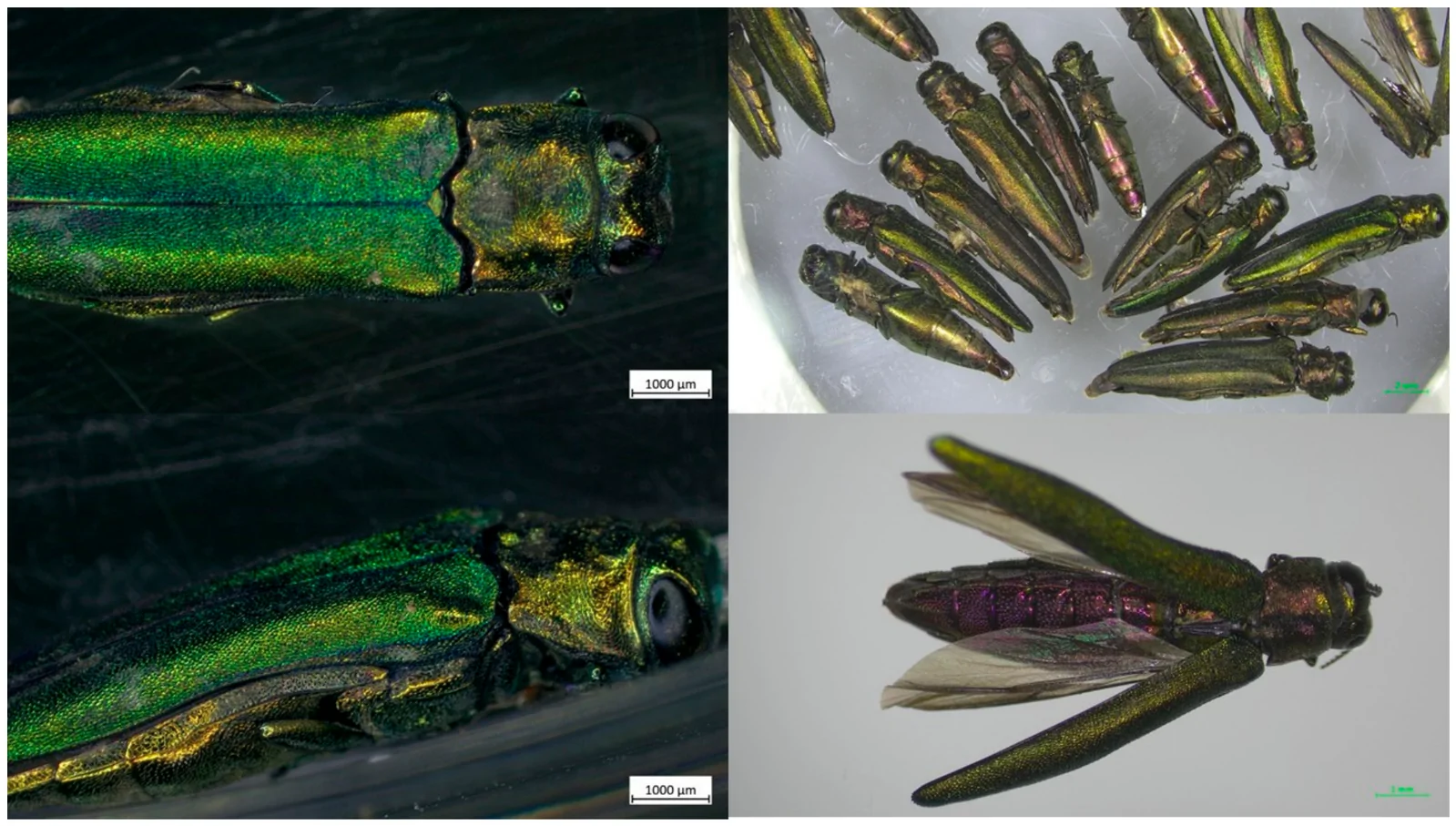
Laboratory-confirmed *A. planipennis* adults from the first positive pheromone-trap catch (photo: Kristína Darnadyová).

**Figure 4:**
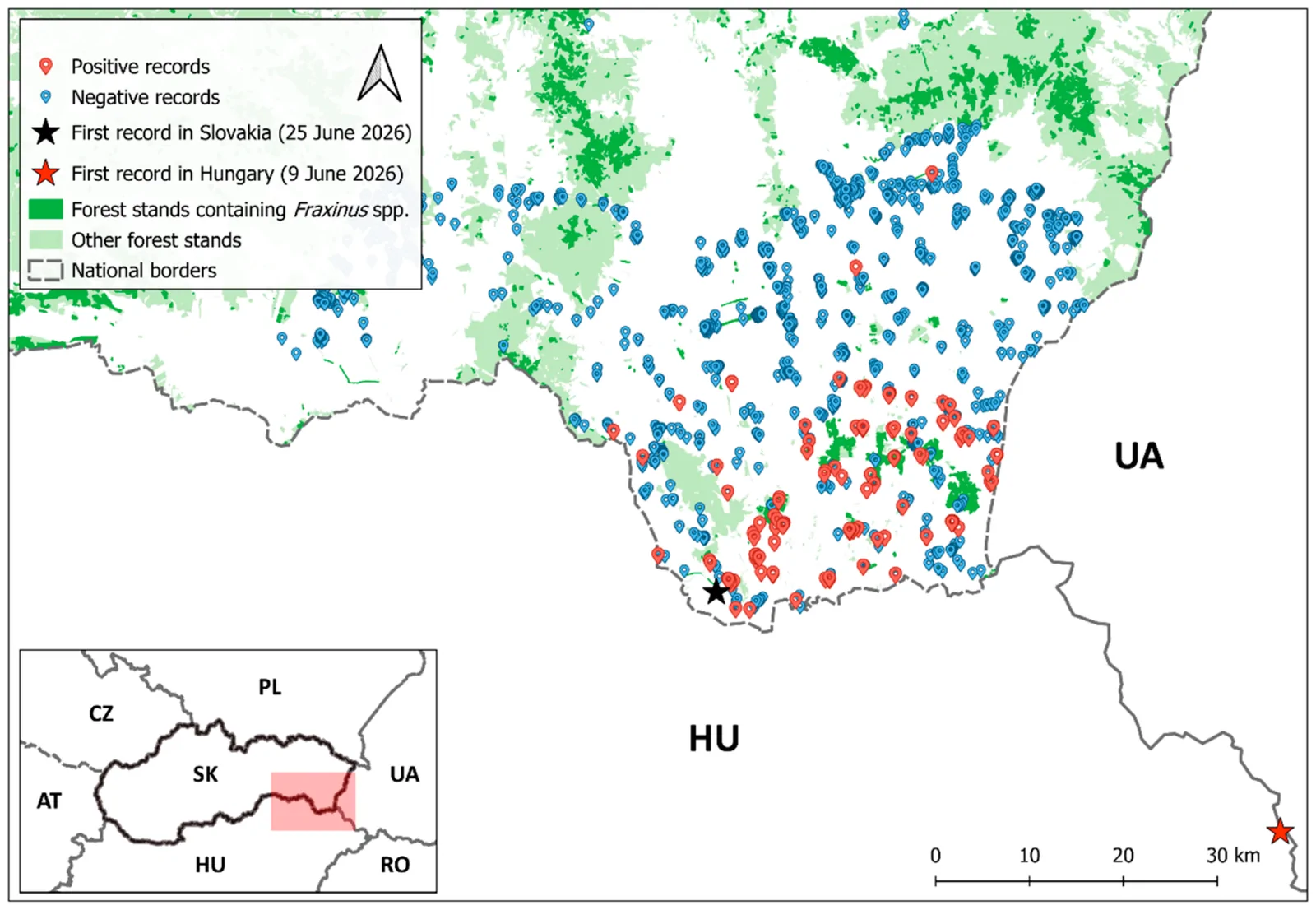
Spatial distribution of positive and negative *A. planipennis* survey records in eastern Slovakia and adjacent border regions and location of the first records of *A. planipennis* in Slovakia and Hungary. Red points indicate positive survey records and blue points indicate negative records; black and red stars mark the first records in Slovakia and Hungary, respectively.

**Figure 5:**
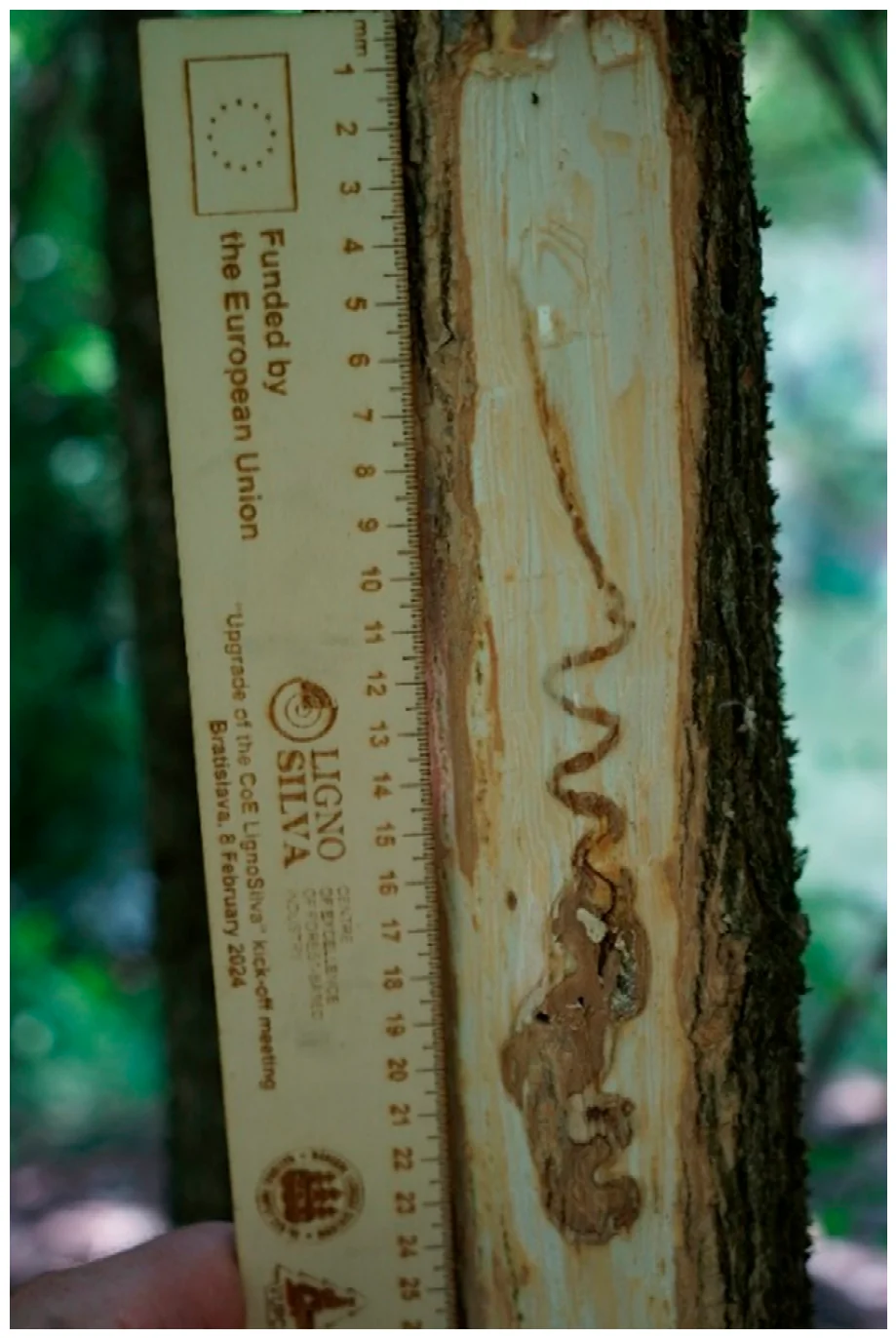
Characteristic galleries of *A. planipennis* (photo: Andrej Gubka).

**Figure 6:**
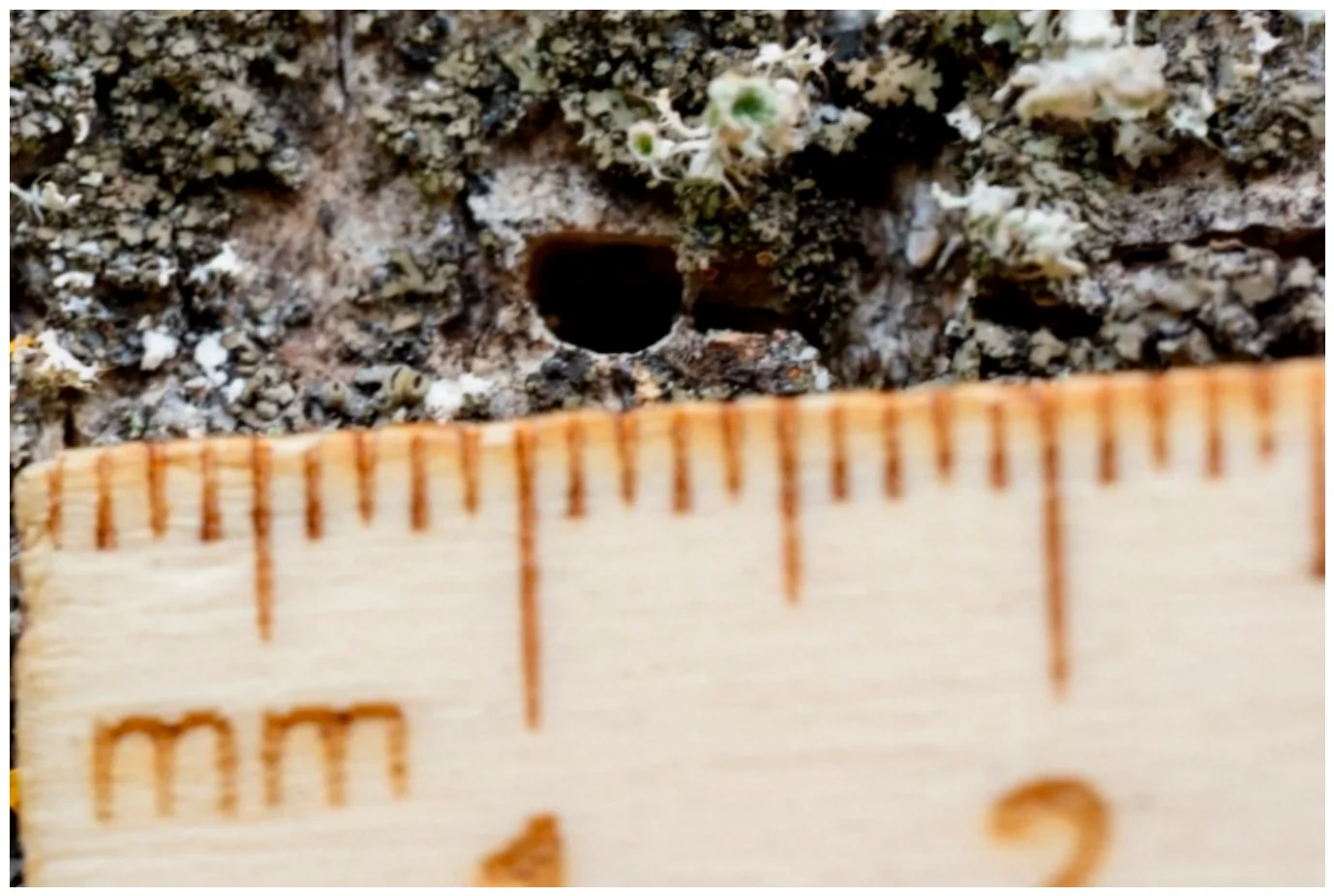
D-shaped exit holes recorded during the delimiting survey (photo: Christo Nikolov).

**Figure 7:**
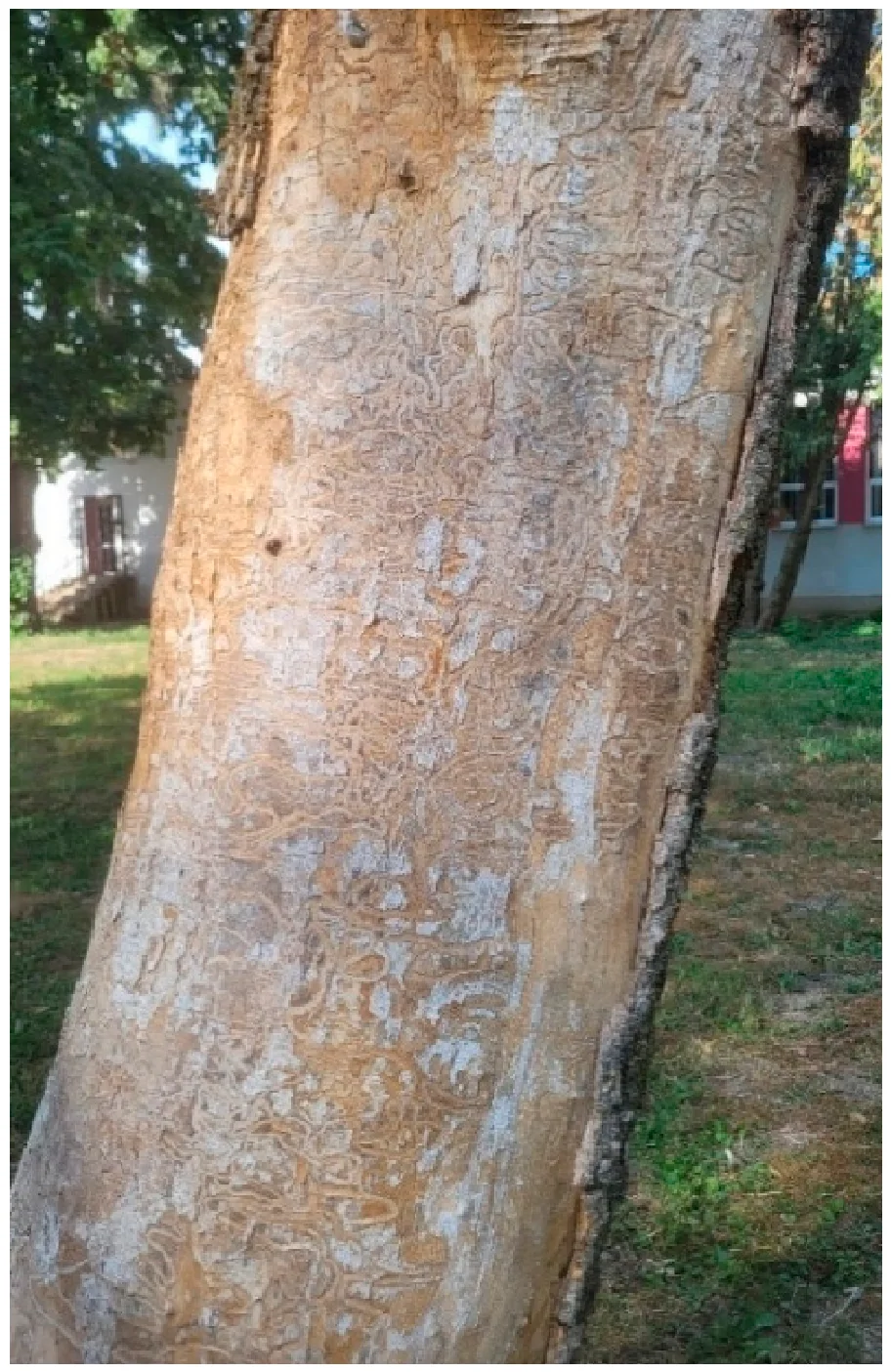
Heavily infested tree with numerous old *A. planipennis* galleries, **consistent with establishment of EAB before its detection in 2026.** (photo: Andrej Gubka).

**Figure 8:**
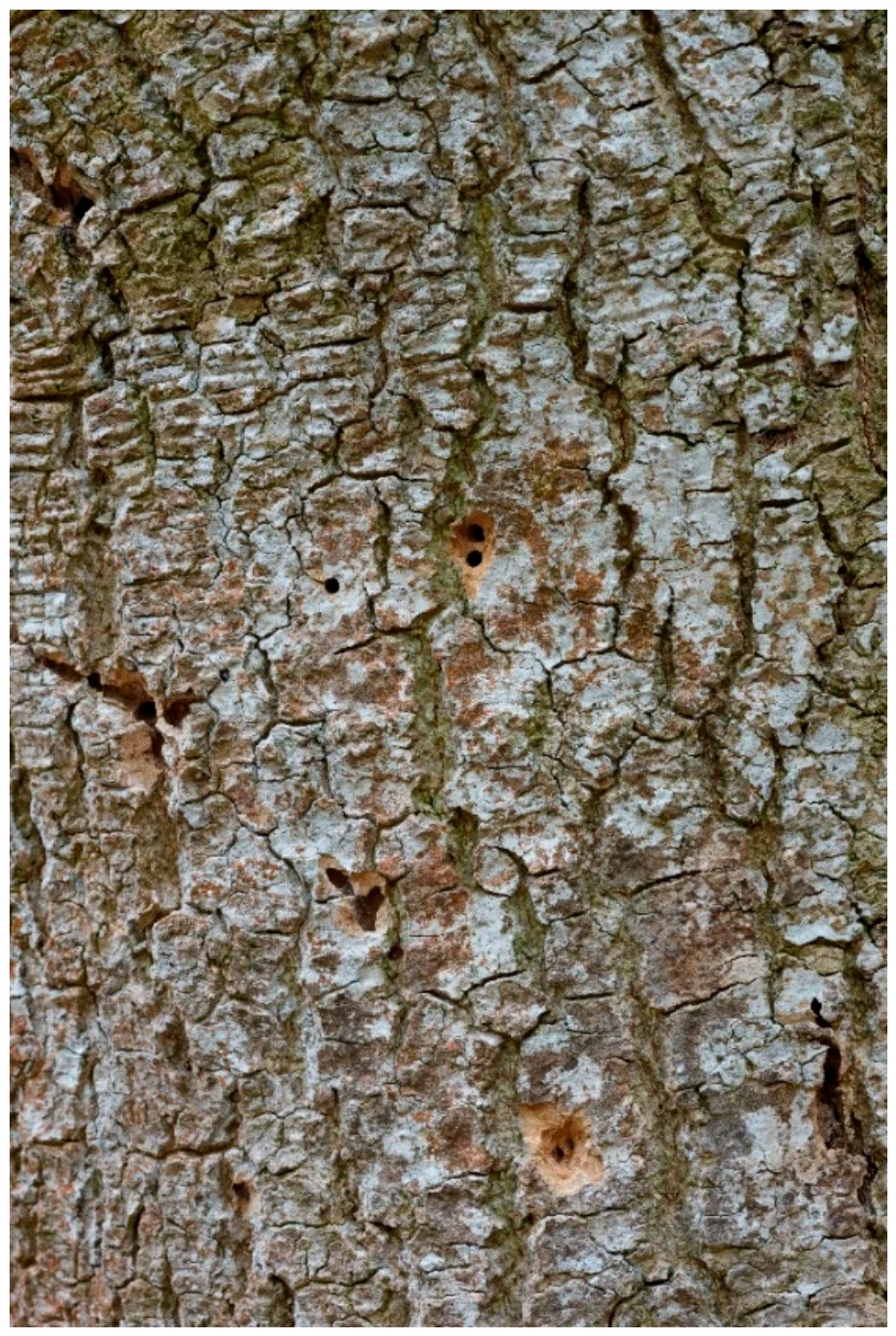
Round exit holes caused by *Hylesinus crenatus* (Fabricius, 1787), similar in size to those of *A. planipennis* and liable to be misidentified without expert assessment (photo: Christo Nikolov).

**Table 1:** Results of the delimiting surveys for *Agrilus planipennis* conducted in Slovakia in July 2026.

Survey Period	Inspected Sites	Positive Sites	Negative Sites	Positive Sites (%)
14–17 July	573	107	466	18.7
20–24 July	1457	26	1431	1.8
27–31 July	220	16	204	7.3
Total	2250	149	2101	6.6

## Data Availability

The spatial distribution of the survey results is presented in this article at the level shown in Figure 4. The underlying survey dataset, including individual records and precise geographic coordinates, is not publicly available because it contains phytosanitary surveillance information subject to applicable data-management requirements.

## References

[1] Baranchikov Y., Mozolevskaya E., Yurchenko G., Kenis M. (2008). Occurrence of the emerald ash borer, *Agrilus planipennis*, in Russia and its potential impact on European forestry. EPPO Bull..

[2] Herms D.A., McCullough D.G. (2014). Emerald ash borer invasion of North America: History, biology, ecology, impacts, and management. Annu. Rev. Entomol..

[3] Baranchikov Y.N., Seraya L.G., Grinash M.N. (2014). All European ash species are susceptible to emerald ash borer *Agrilus planipennis* Fairmaire (Coleoptera: Buprestidae)—A Far Eastern invader. Sib. J. For. Sci..

[4] Orlova-Bienkowskaja M.J., Drogvalenko A.N., Zabaluev I.A., Sazhnev A.S., Peregudova E.Y., Mazurov S.G., Komarov E.V., Struchaev V.V., Martynov V.V., Nikulina T.V. (2020). Current range of *Agrilus planipennis* Fairmaire, an alien pest of ash trees, in European Russia and Ukraine. Ann. For. Sci..

[5] Cipollini D. (2015). White fringetree as a novel larval host for emerald ash borer. J. Econ. Entomol..

[6] Orlova-Bienkowskaja M.J., Bieńkowski A.O. (2016). The life cycle of the emerald ash borer *Agrilus planipennis* in European Russia and comparisons with its life cycles in Asia and North America. Agric. For. Entomol..

[7] EPPO *Agrilus planipennis* (AGRLPL)—EPPO Datasheet. https://gd.eppo.int/taxon/AGRLPL/datasheet.

[8] Siegert N.W., McCullough D.G., Williams D.W., Fraser I., Poland T.M., Pierce S.J. (2010). Dispersal of *Agrilus planipennis* (Coleoptera: Buprestidae) from discrete epicenters in two outlier sites. Environ. Entomol..

[9] Schans J., Schrader G., Delbianco A., Graziosi I., Vos S., EFSA (2020). Pest survey card on *Agrilus planipennis*. EFSA Support. Publ..

[10] European Commission (2019). Commission Delegated Regulation (EU) 2019/1702 of 1 August 2019 supplementing Regulation (EU) 2016/2031 by establishing the list of priority pests. Off. J. Eur. Union.

[11] European Commission (2019). Commission Implementing Regulation (EU) 2019/2072 of 28 November 2019 establishing uniform conditions for the implementation of Regulation (EU) 2016/2031. Off. J. Eur. Union.

[12] EPPO (2007). First report of *Agrilus planipennis* in the region of Moscow, Russia. EPPO Reporting Service.

[13] Straw N.A., Williams D.T., Kulinich O.A., Gninenko Y.I. (2013). Distribution, impact and rate of spread of emerald ash borer *Agrilus planipennis* (Coleoptera: Buprestidae) in the Moscow region of Russia. Forestry.

[14] Musolin D.L., Kirichenko N.I., Karpun N.N., Aksenenko E.V., Golub V.B., Kerchev I.A., Mandelshtam M.Y., Vasaitis R., Volkovitsh M.G., Zhuravleva E.N. (2022). Invasive Insect Pests of Forests and Urban Trees in Russia: Origin, Pathways, Damage, and Management. Forests.

[15] Drogvalenko A.N., Orlova-Bienkowskaja M.J., Bieńkowski A.O. (2019). Record of the emerald ash borer (*Agrilus planipennis*) in Ukraine is confirmed. Insects.

[16] Zviagintsev V.B., Kirichenko N.I., Chernik M.I., Seraya L.G., Baranchikov Y.N. (2025). The emerald ash borer *Agrilus planipennis* Fairmaire (Coleoptera: Buprestidae) invaded Belarus. Acta Biol. Sib..

[17] EPPO (2026). First report of *Agrilus planipennis* in Hungary. EPPO Reporting Service.

[18] Atlas Forest Pests. https://www.forestpests.eu/pest/agrilus-planipennis.

[19] Atlas Škodcovia Drevín. https://www.skodcoviadrevin.sk/skodca/krason-jasenovy-agrilus-planipennis.

[20] (2025). Národný Pohotovostný Plan pre Prípad Podozrenia a Výskytu Prioritného Škodcu Agrilus planipennis.

[21] Ryall K.L., Fidgen J.G., Turgeon J.J. (2011). Detectability of the emerald ash borer (Coleoptera: Buprestidae) in asymptomatic urban trees by using branch samples. Environ. Entomol..

[22] Siegert N.W., McCullough D.G., Liebhold A.M., Telewski F.W. (2014). Dendrochronological reconstruction of the epicentre and early spread of emerald ash borer in North America. Divers. Distrib..

[23] Nageleisen L.-M. (2010). Les Scolytes du Frêne.

[24] Jurc M., Pavlin R., Borkovič D., Pintarič S. (2024). Material for the Beetle Fauna (Coleoptera) of Slovenia, 6. Contribution: Polyphaga: Curculionidae: Scolytinae, Platypodinae.

[25] Kádasi Horáková M., Barta M., Adamčíková K., Ostrovský R., Pastirčáková K. (2023). *Hymenoscyphus fraxineus* on *Fraxinus excelsior* in Slovakia: Distribution and mating types. Biologia.

[26] Skovsgaard J.P., Wilhelm G.J., Thomsen I.M., Metzler B., Kirisits T., Havrdová L., Enderle R., Dobrowolska D., Cleary M., Clark J. (2017). Silvicultural strategies for *Fraxinus excelsior* in response to dieback caused by *Hymenoscyphus fraxineus*. Forestry.

